# Injection of the Stomach in Chronic Gastritis

**Published:** 1850-09

**Authors:** M. M. Rodgers

**Affiliations:** Rochester


### *Injection of the Stomach in Chronic Gastritis.*

MR. EDITOR: *Sir,*—I am now making an experiment, that, so far as I know, is new. I am treating a case of chronic gastritis by injecting the stomach with a solution of the nitrate of silver. I began with one grain, of the strongest, to one ounce of water. I intend to increase the strength as long as the patient can bear it; it does well so far. I introduce it by means of the stomach-pump,—I pump the stomach full, and after it remains a few minutes, pump it out again. This, I think, is new—but it must prove serviceable. Will you please give this a corner in your journal? Yours, respectfully,

M. M. RODGERS, M. D.

ROCHESTER, August, 1850.

